# Genotype by environment interaction and stability analysis for harvest date in sugar beet cultivars

**DOI:** 10.1038/s41598-024-67272-7

**Published:** 2024-07-11

**Authors:** Saeed Sadeghzadeh Hemayati, Farahnaz Hamdi, Ali Saremirad, Hamze Hamze

**Affiliations:** 1 Sugar Beet Seed Institute (SBSI), Agricultural Research, Education and Extension Organization (AREEO), Karaj, Iran; 2 Sugar Beet Research Department, Hamedan Agricultural and Natural Resources Research and Education Center, AREEO, Hamedan, Iran

**Keywords:** Adaptation, Growth period, Selection, Sugar content, Plant breeding, Plant development

## Abstract

This research assessed the quantitative and qualitative reactions of commercially grown sugar beets to four different harvest dates and their yield stability. The study followed a split-plot design within a randomized complete block design over 3 years. The main plot involved 10 sugar beet cultivars, while the subplot involved four harvest dates: August 13 (HD_1_), September 7 (HD_2_), October 3 (HD_3_), and November 12 (HD_4_). The study found that environmental conditions, genotypes, and harvest dates significantly affected various traits of sugar beet. Yearly environmental variations and their interactions with genotypes and harvest dates had substantial impacts on all measured traits at the 1% probability level. Additive main effect and multiplicative interaction analysis based on white sugar yield indicated that genotype and environment's additive effects, as well as the genotype–environment interaction, were significant at 1% probability level. Shokoufa and Arya, which exhibit high white sugar yield (WSY) and low first interaction principal component (IPC_1_) values, are identified as desirable due to their stability across different environments. Among the harvest dates in different years, the fourth and third dates showed a higher yield than the total average. Perfekta and Ekbatan exhibited high specific adaptability. According to the multi-trait stability index, Arta, Arya and Sina were recognized as stable and superior across all measured traits.

## Introduction

According to United Nations^1^ projections, the global population is anticipated to rise from 7.7 billion in 2019 to 8.5 billion in 2030, 9.7 billion in 2050, and 10.9 billion in 2100. With this population growth, the demand for food is expected to increase significantly^2^. Sugar, a vital nutrient throughout history, acts as a tonic substance and contributes a substantial portion of energy in the human diet^3^. As an essential agricultural product, sugar beet is exclusively utilized in the sugar industry^4^ and ranks as one of the principal sources of sugar production following sugar cane^5,6^. Currently, it is estimated to contribute to 20–30% of global sugar production^5–7^. While sugar stands as the primary product of sugar beet, the plant also yields various by-products such as molasses, pomace, and ethyl alcohol during the sugar production process^8^. Furthermore, the plant's leaves contain protein compounds^9,10^ and balanced amino acids^4,11^, highlighting the nutritional quality of sugar beet leaves. Given the significance of sugar beet in human nutrition, it is imperative to focus on both the quantitative and qualitative aspects of its production.

Sugar beet yield formation is intricately linked to weather conditions^12,13^. Temperature influences canopy development during the spring, while water availability can constrain growth in the summer. Prolonging the growth phase can lead to increased yield potential^14^. Timely canopy formation enhances light absorption, particularly during periods of high irradiance in May and June, boosting biomass production and ultimately contributing to higher sugar beet yields^12^. Efficient crop management practices, as highlighted by Finkenstadt^15^, show a positive correlation between the sugar beet's root yield (RY) potential and the amount of intercepted radiation during sowing and harvesting.

Harvest timing critically affects the yield and quality of sugar beet crops^16^. Certain genotypes with high sugar content (SC) are ideal for early harvesting. Delaying harvest by 27 days can increase RY by 11.35 t ha^−1^ and WSY by 1.69 t ha^−1^^17^. Optimal harvest time for maximum sucrose, RY, and sugar yield (SY) is 210 days from sowing^18^. Harvesting at this time generally improves most traits, except for sugar lost to molasses and *alpha amino* nitrogen (N)^19^.

It is crucial to select sugar beet genotypes with high potential yield and implement agronomic practices that are well-suited and synchronized with the plant’s requirements and needs^20^. The most significant commercial trait of sugar beets is WSY, which is strongly affected by the environment and highly correlated with RY and SC^21^. The interaction between genetic composition and environmental factors plays a critical role in determining plant growth, development, and yield. Genotypes in crop production exhibit varying outcomes and rankings due to genotype-by-environment interaction (GEI)^22^. Plants experience diverse inputs and stimuli from the environment, including water, nutrients, and radiation^23^. The genetic concepts of adaptation and stability are often associated and elucidated within the framework of GEI^24^. Various statistical methods, such as regression analysis, nonparametric statistics, and multivariate models, are employed to explore and assess different genotypes. Recently, Olivoto, et al.^25^ presented the theoretical foundation of the multi-trait stability index (MTSI) for selecting high-yield and stable genotypes in multi environment trials (METs) based on multiple traits, considering both fixed and random effects models. The MTSI is computed by assessing the distance from the ideal genotype, estimated through factor analysis. This index allows for the selection of stable genotypes with a positive selection differential for traits intended to increase and a negative selection differential for traits intended to decrease. Moreover, this stability index is beneficial for breeders aiming to simultaneously select for average performance and stability by considering several traits, offering a distinct selection process that is easy to interpret and considers the correlation structure among traits^25^.

Additive main effects and multiple interactions (AMMI) analysis is commonly used to interpret genotypic responses to the environment and GEI^26^. AMMI integrates analysis of variance and principal component analysis (PCA) methods^27^. In the final stage, AMMI eliminates the additive effects from the interaction using analysis of variance and scrutinizes the interaction structure using the PCA method. The AMMI model, often used in conjunction with the genotype and the genotype-by-environment (GGE) biplot graphical model, aims to identify mega-environments (MGEs) and winning genotypes within each MGE. This graphical model, founded on PCA^28–31^, facilitates the straightforward evaluation of genotype stability and the integration of stability with genotype yield across different environments through the graphical representation of GEI. An essential aspect of the GGE biplot is its capacity to determine which genotype holds the greatest potential in a specific environment or subgroup^32^.

The study was designed to investigate the impact of different harvest dates on the quantitative and qualitative characteristics of sugar beet cultivars and the stability of these cultivars across various harvest times. While past studies^33–39^ have concentrated on cultivar stability across different years and locations, research on cultivar stability across different harvest dates has been limited. Therefore, identifying a cultivar with high yield stability under diverse environmental conditions would confer a significant advantage.

## Materials and methods

### Plant materials and experimental conditions

The study encompassed nine cultivars developed at the Sugar Beet Seed Institute (SBSI), Alborz, Iran, and one foreign cultivar (Perfekta) (Table 1). Field trials were executed at the Motahhari Sugar Beet Research Station (located at 50° 52′ E, 35°50′ N, and an elevation of 1244 m above sea level) in Karaj, Alborz, Iran over three cropping seasons (2019–2021). Karaj experiences a cold temperate climate, with an average annual temperature of 14.4 °C and annual rainfall of 247.3 mm. The region is semi-arid, characterized by cold winters and partly mild summers (Table 2). The research was designed as a split plot experiment utilizing a randomized complete block design with four replications. The experiment featured 10 commonly grown sugar beet cultivars in Iran and four harvest dates (August 13, September 7, October 3, and November 12). The primary plot size measured 12 m^2^, comprising three 8-m-long rows spaced 0.5 m apart. The soil was classified as clay-loam, with a pH of 7.5. Sowing was conducted using a seed planter on March 15 across all 3 years. Standard agronomic practices were implemented for the sugar beets during the growing season. The battle against broad-leaved and narrow-leaved weeds in the fields was conducted through manual weeding. To monitor and prevent infestations of pests and diseases in sugar beets, regular field inspections were carried out. During these inspections, no pests or diseases were detected.

**Table 1:** The roster of sugar beet cultivars.

Row	Cultivar	Characteristics	Row	Cultivar	Characteristics
1	Arta	Resistant to rhizomania and cyst nematode	6	Paya	Tolerant to drought stress
2	Arya	Resistant to rhizomania and cyst nematode	7	Perfekta	Resistant to rhizomania
3	Asia	Resistant to rhizomania and cyst nematode	8	Sharif	Resistant to bolting
4	Ekbatan	Resistant to rhizomania and rhizoctonia	9	Shokoufa	Resistant to rhizomania and rhizoctonia
5	Motahhar	Resistant to rhizomania	10	Sina	Resistant to rhizomania, rhizoctonia

**Table 2:** The average temperatures and rainfall data recorded at the research stations for the years 2019, 2020, and 2021.

Month	Average temperature (°C)			Rainfall (mm)		
	2019	2020	2021	2019	2020	2021
January	6.00	4.10	5.20	42.80	57.20	28.10
February	7.20	7.50	9.90	20.60	1.40	84.60
March	10.50	12.90	13.30	3.80	101.60	25.40
April	15.50	15.20	22.00	46.10	32.00	85.40
May	24.10	24.30	25.20	7.20	11.70	20.90
June	30.50	29.50	31.90	0.00	0.00	0.00
July	33.00	31.10	32.20	0.00	0.00	0.30
August	30.50	28.70	30.90	0.90	0.00	0.00
September	26.60	25.70	28.20	22.40	30.00	20.30
October	19.50	18.30	18.40	56.00	75.20	8.40
November	9.40	11.40	11.00	25.70	11.00	64.40
December	7.80	5.00	7.80	29.00	28.40	54.80

### Measurement of quantitative and qualitative traits

After harvesting and recording the RY, the roots were washed, and a brei sample was randomly extracted from the roots of each plot. Subsequently, the collected samples were stored in a freezer at − 18 °C. 26 g of frozen samples were then mixed with 177 ml of lead (II) hydroxide acetate for 3 min using a mixer. The resulting solution was filtered through a sieve to obtain a clear liquid, which was then analyzed using the Betalyser device to quantify elements such as SC, N, sodium (Na^+^), and potassium (K^+^) for the evaluation of sugar beet quality^40^. The resulting values was utilized to estimate WSY, utilizing Eqs. (1) to (3), respectively^41,42^. However, all international, national and institutional guidelines^43^ have been taken into account in various stages of experiments.

1 $$ MS = 0.343\left( {K^{ + } + Na^{ + } } \right) + 0.094\left( {alpha\;amino\;N} \right) - 0.31 $$

2 $$ WSC = SC - \left( {MS + 0.6} \right) $$

3 $$ WSY = WSC \times RY $$

where MS is molasses sugar (%), K^+^ is potassium (meq.100 g^−1^), Na^+^ is sodium (meq.100 g^−1^), *alpha-amino-*N is nitrogen (meq.100 g^−1^), WSC is white sugar content (%), SC is sugar content (%), WSY is white sugar yield (t ha^−1^) and RY is root yield (t ha^−1^).

### Statistical analysis

Prior to conducting any analysis, the Grubbs test^44^ was utilized to assess the data, assuming normality. Additionally, the Bartlett test^45^ was employed to examine the uniformity of experimental error variances across different years. Upon confirming the consistency of these error variances, a combined analysis of variance was executed. This analysis was conducted with a random year effect, while assuming the cultivar and harvest date effects to be fixed. The MTSI was calculated to determine the stability of RY, SC, K^+^, Na^+^ and N based on Eq. 4^46^.

4 $$ MTSI_{i} = \left[ {\mathop \sum \limits_{j = 1}^{f} \left( {\gamma_{ij} - \gamma_{j} } \right)^{2} } \right]^{0.5} $$

where $MTSI_{i}$ is the multi-trait stability index of the genotype *i*, $\gamma_{ij}$ is the score of the genotype *i* in the factor *j*, and $\gamma_{j}$ is the score of the ideal genotype in the factor *j*. Scores were calculated based on factor analysis for genotypes and traits.

Due to its incorporation of other studied traits, WSY is deemed a pivotal and ultimate trait. Consequently, an AMMI stability analysis was conducted with regard to this trait. Equation 5, as outlined in the AMMI method^26^, was employed to carry out the stability analysis in 12 environments (2 years and four harvest dates).

5 $$ Y_{ge} = \mu + \alpha_{g} + \beta_{e} + \mathop \sum \limits_{n} \lambda_{n} \gamma_{gn} \delta_{en} + \rho_{ge} $$

where $$Y_{ge} $$ is the yield of genotype *g* in environment *e*; $$\mu$$ is the grand mean; $$\alpha_{g}$$ is the genotype deviation from the grand mean; $$\beta_{e}$$ is the environment deviation; $$\lambda_{n}$$ is the singular value for interaction principal component (*IPC*_*n*_) and correspondingly $$\lambda_{n}^{2}$$ is its eigenvalue; $$\gamma_{gn}$$ is the eigenvector value for genotype *g* and component *n*; $$\delta_{en}$$ is the eigenvector value for environment *e* and component *n*, with both eigenvectors scaled as unit vectors; and $$\rho_{ge}$$ is the residual. Using the AMMI analysis of variance in SAS software version 9.4^47^, the eigenvalues for each cultivar and environment were calculated. Biplots of this model were drawn to determine the general and specific adaptability of the cultivars. During this investigation, 13 statistics derived from the AMMI model were employed to identify the stable cultivar, through Eqs. (6) to (18).

6 $$ ASTAB = \mathop \sum \limits_{n = 1}^{{N^{\prime}}} \lambda_{n} \gamma_{in}^{2} $$

7 $$ ASI = \sqrt {\left[ {PC_{1}^{2} \times \theta_{1}^{2} \left] + \right[PC_{2}^{2} \times \theta_{2}^{2} } \right]} $$

8 $$ ASV = \sqrt {\left[ {\frac{{{\text{SS}}_{{{\text{IPCA}}1}} }}{{{\text{SS}}_{{{\text{IPCA}}2}} }}\left( {IPCA_{1} } \right)} \right]^{2} + \left( {{\text{IPCA}}_{2} } \right)^{2} } $$

9 $$ AV_{AMGE} = \mathop \sum \limits_{j = 1}^{E} \mathop \sum \limits_{n = 1}^{{N^{\prime}}} \left| {\lambda_{n} \gamma_{in} \delta_{jn} } \right| $$

10 $$ Da = \sqrt {\mathop \sum \limits_{n = 1}^{N} \left( {\lambda_{n} \gamma_{in} } \right)^{2} } $$

11 $$ D_{Z} = \mathop \sum \limits_{n = 1}^{{N^{\prime}}} \gamma_{in}^{2} $$

12 $$ EV = \mathop \sum \limits_{n = 1}^{N} \frac{{\gamma_{in}^{2} }}{n} $$

13 $$ FA = \mathop \sum \limits_{n = 1}^{{N^{\prime}}} \lambda_{n}^{2} \gamma_{in}^{2} $$

14 $$ MASI = \sqrt {\mathop \sum \limits_{n = 1}^{N^{\prime}} {\text{PC}}_{{\text{n}}}^{2} \times \theta_{1}^{2} } $$

15 $$ MASV = \sqrt {\mathop \sum \limits_{n = 1}^{N^{\prime}} \left( {\frac{{SSIPC_{n} }}{{SSIPC_{n + 1} }}} \right) \times \left( {PC_{n} } \right)^{2} + \left( {PC_{{N^{\prime}}} } \right)^{2} } $$

16 $$ SIPC_{i} = \mathop \sum \limits_{n = 1}^{N} \left| {\lambda_{n}^{0.5} \gamma } \right| $$

17 $$ Z_{a} = \mathop \sum \limits_{n = 1}^{{N^{\prime}}} \left| {\theta_{n} \gamma_{in} } \right| $$

18 $$ WAAS_{i} = \frac{{\mathop \sum \nolimits_{k = 1}^{P} \left| {IPCA_{ik} \times EP_{k} } \right|}}{{\mathop \sum \nolimits_{k = 1}^{P} EP_{k} }} $$

where ASTAB is AMMI-based stability parameter^48^, ASI is the AMMI stability index^49^, ASV is the AMMI stability value (where ${\text{SS}}_{{{\text{IPCA}}1}}$ is the sum of squares for IPCA_1_, ${\text{SS}}_{{{\text{IPCA}}2}}$ is the sum of squares for IPCA_2_, and the IPCA_1_ and IPCA_2_ scores are the genotype scores in the AMMI model)^50^, AV_AMGE_ is the sum across environments of the absolute value of GEI modeled by AMMI^51^, Da is Annicchiarico’s D parameter^52^, D_Z_ is Zhang’s D parameter^53^, EV is average of the squared eigenvector values^54^, FA is stability measure based on fitted AMMI model^55^, MASI is modified AMMI stability index^56^, MASV is modified AMMI stability value (where, $SSIPC_{n}$ is the sum of squares of the nth IPC and PC_n_ is the scores of nth IPC)^51^, SIPC is sum of the absolute values of the IPC scores^57^, and Za is the absolute value of the relative contribution of IPCAs to the interaction^51^, WAAS is the weighted average of absolute scores^25^.

## Results

### Combined analysis of variance

Variance analysis revealed significant impacts of various factors on the traits measured in this study, including WSY, RY, SC, Na^+^, K^+^, and N (Table 3). The results indicated that the year had a significant effect on all traits at the 1% probability level. The main effect of cultivar showed a significant impact on SC at the 5% probability level and K^+^ at the 1% probability level. Furthermore, the interaction between year and cultivar significantly affected all traits at the 1% probability level. The main effect of harvest date was significant for WSY, and RY at the 5% probability level. However, its two-way interaction with the environmental conditions of the year and its three-way interaction involving cultivar and environmental conditions had significant effects on all traits at the 1% probability level.

**Table 3:** Combined analysis of variance of sugar beet yield and quality parameters in Karaj (2019–2021).

Source of variation	df	Mean of squares					
		White sugar yield	Root yield	Sugar content	Na^+^	K^+^	*Alpha amino* N
Year	2	280.13**	4173.82**	127.77**	92.05**	85.06**	38.35**
Replication (Year)	6	1.65	177.39	3.45	0.99	0.84	0.34
Cultivar	9	21.47^ns^	417.19^ns^	14.49*	19.33^ns^	5.59 **	1.67^ns^
Year-cultivar	18	17.78**	669.39**	4.21**	9.43**	1.19**	1.85**
E_(a)_	54	1.06	81.13	0.63	0.36	0.09	0.12
Harvest date	3	391.91*	24,589.57*	99.89^ns^	2.97^ns^	0.60^ns^	8.27^ns^
Year-harvest date	6	50.23**	4785.89**	33.95**	7.69**	6.55**	6.36**
Cultivar-harvest date	27	2.62^ns^	172.24^ns^	1.04^ns^	1.02^ns^	0.43^ns^	0.42^ns^
Year-cultivar-harvest date	54	2.53**	220.47**	1.59**	1.13**	0.52**	0.32**
E_(b)_	180	1.31	99.85	0.46	0.23	0.10	0.11
Coefficient of variation (%)		15.91	14.62	4.91	14.72	6.23	17.40

**1% probability level of significance; *5% probability level of significance; ns: non-significant.

### Stability analysis

The MTSI was employed to comprehensively evaluate experimental genotypes across various independent traits, including RY, SC, Na^+^, K^+^, and N, facilitating the identification of desirable cultivars. The factor decomposition was based on PCA, followed by Varimax rotation for result interpretation. Table 4 presents the factor analysis outcomes, selecting factors with eigenvalues greater than one. Each factor's variance is expressed as a percentage, reflecting its significance in explaining overall data variations. Two independent factors accounted for 65.70% of the total data variance in this analysis.

**Table 4:** Factor analysis using principal component analysis: eigenvalues, factor coefficients, relative and cumulative variance, and varimax rotation (Karaj, 2019–2021).

Traits	Factors		Communality	Uniqueness’s
	Factor 1	Factor 2		
Root yield	0.04	− 0.74	0.55	0.45
Sugar content	− 0.93	− 0.02	0.87	0.14
Na^+^	0.76	− 0.49	0.82	0.18
K^+^	− 0.06	− 0.73	0.54	0.46
*Alpha amino* N	− 0.61	− 0.38	0.51	0.49
Eigenvalue	1.85	1.43	–	–
Relative Variance (%)	37.10	28.60	–	–
Cumulative variance (%)	37.10	65.70	–	–

**Figure 1:**
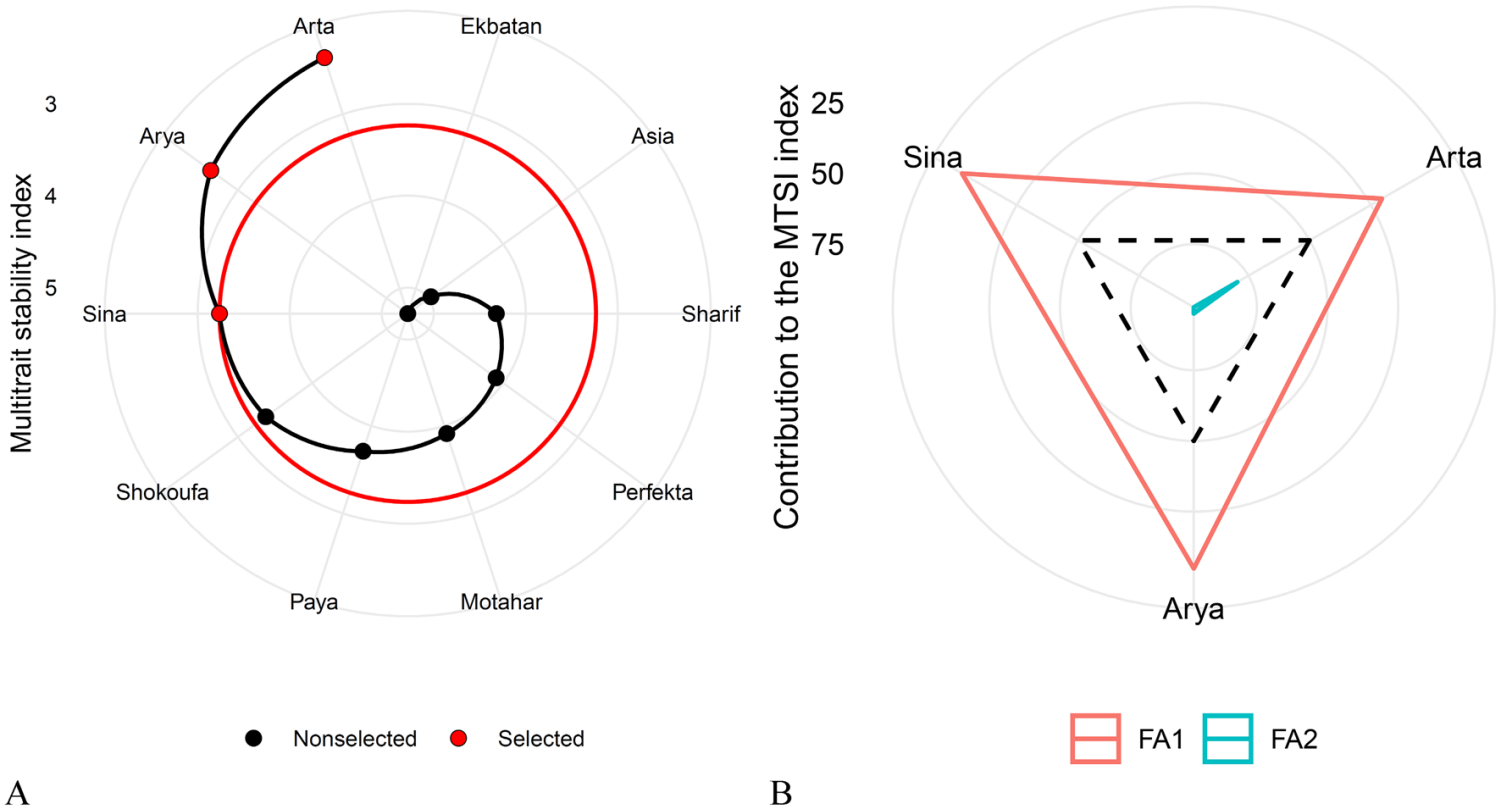
Ranking of cultivars in ascending order based on the multitrait stability index (**A**) and strengths and weaknesses of selected cultivars as the ratio of each factor in the calculated multitrait stability index (**B**).

The first factor, with an eigenvalue of 1.85, explained 37.10% of the total variance. It exhibited hight negative factor coefficients for SC and N, and a positive coefficient for Na^+^. The second factor, with an eigenvalue of 1.43 and explaining 28.60% of the variations, included hight negative factor coefficients for RY and K^+^. The MTSI index for the studied cultivars was calculated based on the factor scores of these two factors. According to this index, a lower MTSI value indicates a closer proximity to the ideal cultivar, while a higher MTSI value suggests a greater distance from the ideal cultivar, making it less desirable. In Fig. 1A, the experimental cultivars are ranked from the highest to the lowest MTSI value. The cultivar with the highest MTSI value is positioned at the center, while the cultivar with the lowest MTSI value is placed in the outermost circle. By applying a 30% selection pressure, the Arta cultivar was ranked first, with Arya and Sina identified as the most ideal cultivars in terms of all traits. Figure 1B highlights the strengths and weaknesses of selected cultivars based on each factor's contribution to the MTSI index. In this diagram, a lower factor share (closer to the outer edge) indicates that the attributes within that factor are nearer to the ideal state. The dashed line represents the theoretical value if all factors contributed equally. Cultivars Sina, Arya, and Arta had the lowest values in the first factor for SC, N, and Na^+^, which had the highest factor coefficients in this factor, suggesting they are close to the ideal cultivar. The ideal cultivar is defined by the traits included in each factor and the goals intended to improve those traits. Arta had the lowest share of the second factor, indicating its proximity to the ideal cultivar in terms of RY and K^+^. In other words, this cultivar exhibits high RY and low K^+^ content.

To enhance the reliability of the experimental analysis and to study interactions between main effects more precisely, the data analysis of WSY was conducted using the AMMI model. The AMMI analysis of variance for WSY demonstrated statistically significant differences between cultivars and environments (additive effects) as well as GEI at the 1% probability level (Table 5). The GEI was further analyzed, yielding the first and second interaction principal components (IPC_1_ and IPC_2_), both of which were found to be significant at the 1% probability level. These components accounted for 60.20% and 15.21% of the sum of squares of the GEI effect, respectively. Combined, IPC_1_ and IPC_2_ explained 75.41% of the total GEI variance (Table 5).

**Table 5:** Analysis of variance of white sugar yield in sugar beet cultivars using the AMMI Model in Karaj (2019–2021).

Source	df	Sum of squares	Mean of squares	Relative variance (%)
Environment	11	2037.79	185.21**	–
Error 1	24	48.11	2.00	–
Genotype	9	193.23	21.47**	–
Genotype-environment interaction	99	527.07	5.33**	–
Interaction principal component 1	19	317.484	16.72**	60.20
Interaction principal component 2	17	80.25	4.72**	15.21
Noise	63	129.40	2.05**	24.52
Error 2	216	255.54	1.18	–

**1% probability level of significance; *5% probability level of significance; ns: non-significant.

**Figure 2:**
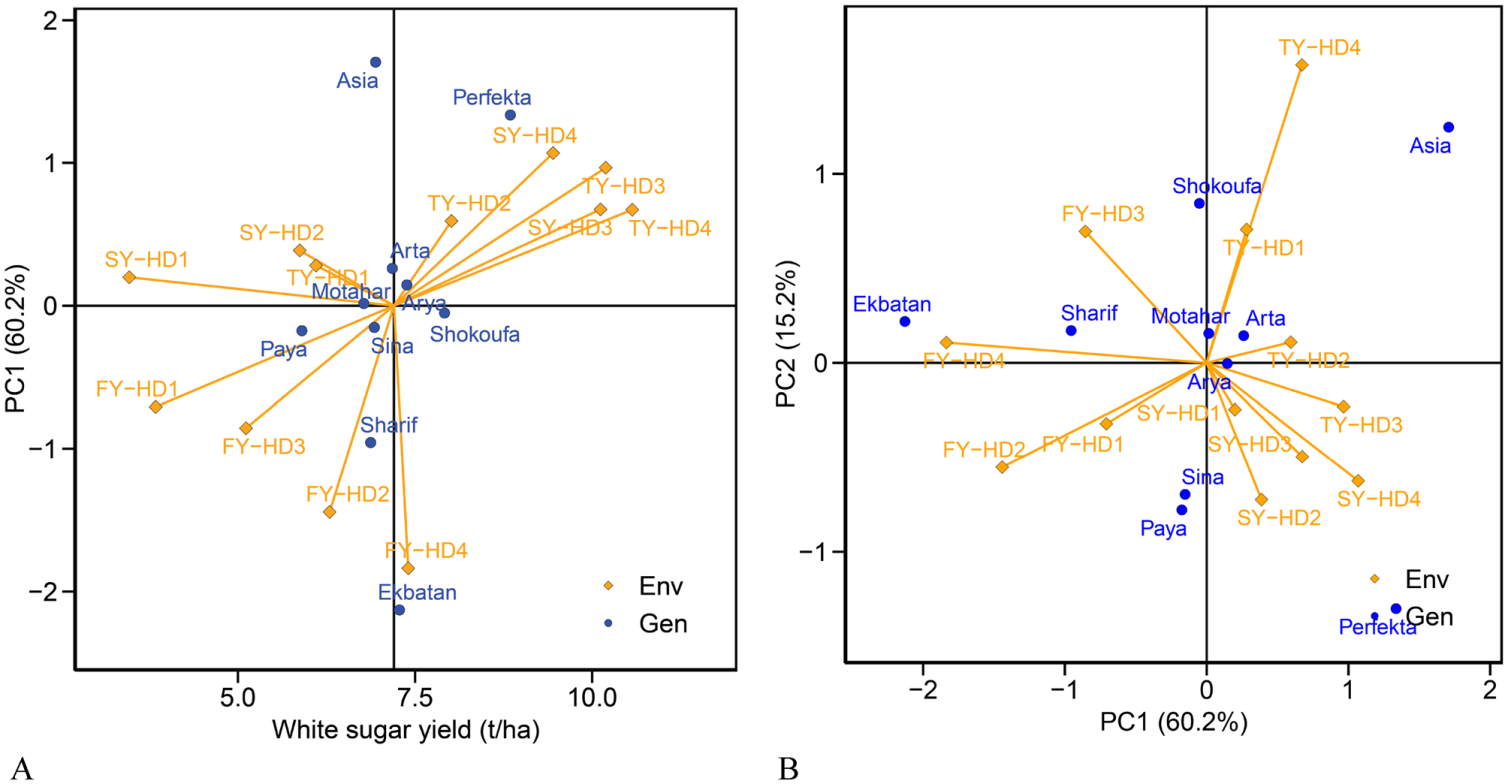
Scatter plot illustrating the relationship between cultivars and environments, using the mean white sugar yield and the first (**A**) and second (**B**) principal components. *FY* first year, *SY* second year, *TY* third year, *HD*_*1*_ harvest dates on August 13, *HD*_*2*_ harvest dates on September 7, *HD*_*3*_ harvest dates on October 3, *HD*_*4*_ Harvest dates on October November 12.

To consider yield stability and specific adaptation of cultivars to environments, WSY biplots with the IPC_1_ (Fig. 2A) and biplots of the first two IPCs (Fig. 2B) were used. According to the biplot of the average WSY against the IPC_1_ of the GEI, cultivars with higher WSY (horizontal axis) and lower values (close to zero) in terms of the IPC_1_ (vertical axis) are more desirable. Based on this analysis, among the cultivars, Shokoufa and Arya were recognized as the most stable cultivars due to their higher-than-average WSY and the low value of the IPC_1_. Conversely, Ekbatan and Perfekta displayed both high positive and negative values of IPC_1_, suggesting lower stability compared to other studied cultivars (Fig. 2A). The results of environments (year-harvest date) demonstrated that the highest WSY was achieved with HD_4_, followed sequentially by HD_3_ and HD_2_, with HD_1_ yielding the lowest WSY (Fig. 2A). These results indicate that a delay in the harvesting schedule leads to a substantial increase in WSY. In Fig. 2B, the biplot values of the first and second IPCs for cultivars and environments are displayed. A total of 75.41% of the variation related to the multiplicative effect was explained by this biplot. According to this biplot, cultivars close to the origin of the coordinates have general adaptability. As seen in the figure, Arya and Motahar are generally adaptable due to their proximity to the origin of the coordinates, indicating less yield fluctuation across different years and harvest dates.

Various stability statistics from the AMMI analysis were calculated and are presented in Table 6, along with the average WSY. The average WSY of cultivars across all environments was estimated to be 7.20 t ha^−1^. Among the cultivars, Perfecta and Shokoufa exhibited the highest WSY, with 8.84 and 7.92 t ha^−1^, respectively. Conversely, the Paya had the lowest WSY, averaging 5.90 t ha^−1^. The WSY of Ekbatan, Arta, and Aria were 7.28, 7.18, and 7.38 t ha^−1^, respectively, aligning closely with the overall average yield.

**Table 6:** Mean white sugar yield and various AMMI stability parameters for sugar beet cultivars across 12 environments in Karaj (2019–2021).

Cultivar	Yield	ASTAB	ASI	ASV	AV_AMGE_	Da	D_Z_	EV	FA	MASI	MASV	SIPC	Za	WAAS
Arta	7.18	0.64	0.16	1.04	4.22	1.65	0.41	0.04	2.74	0.17	1.31	1.38	0.10	0.28
Arya	7.38	0.33	0.09	0.57	3.24	1.14	0.30	0.02	1.29	0.10	0.81	0.90	0.06	0.16
Asia	6.94	4.79	1.04	6.86	17.70	6.25	0.82	0.17	39.11	1.04	7.19	3.65	0.43	1.40
Ekbatan	7.28	4.91	1.28	8.42	20.50	6.94	0.73	0.13	48.10	1.28	8.46	3.00	0.44	1.51
Motahhar	6.78	1.51	0.02	0.16	6.74	2.30	0.66	0.11	5.29	0.09	1.28	1.70	0.07	0.15
Paya	5.90	1.70	0.16	1.04	7.17	2.70	0.64	0.10	7.31	0.18	2.02	2.39	0.14	0.37
Perfekta	8.84	3.63	0.83	5.45	16.19	5.26	0.74	0.14	27.70	0.83	5.87	3.14	0.36	1.15
Sharif	6.87	2.29	0.57	3.79	11.37	3.81	0.68	0.11	14.50	0.58	4.02	2.66	0.25	0.80
Shokoufa	7.92	1.40	0.13	0.87	6.73	2.50	0.57	0.08	6.25	0.15	1.90	1.96	0.11	0.27
Sina	6.93	1.42	0.14	0.92	6.56	2.45	0.59	0.09	6.02	0.16	1.80	2.19	0.13	0.33

*ASTAB* AMMI-based stability parameter, *ASI* AMMI stability index, *ASV* AMMI stability value, *AV*_*AMGE*_ sum across environments of the absolute value of GEI modeled by AMMI, *Da* Annicchiarico’s D parameter, *D*_*Z*_ Zhang’s D parameter, *EV* average of the squared eigenvector values, *FA* stability measure based on fitted AMMI model, *MASI* modified AMMI stability index, *MASV* modified AMMI stability value, *SIPC* sum of the absolute values of the IPC scores, *Za* absolute value of the relative contribution of IPCs to the interaction, *WAAS* weighted average of absolute scores.

The results obtained using several stability statistics—ASTAB, AV_AMGE_, Da, D_Z_, EV, FA, and SIPC—indicated that the Arya and Arta, which had the lowest values for these statistics, were the most stable. In contrast, the Ekbatan and Asia, with the highest values for these statistics, were identified as the most unstable. Additional stability statistics, including ASI, ASV, MASI, MASV, Za, and WAAS, also pointed to Motahar and Arya as the most stable, given their lowest values in these metrics. Similarly, Ekbatan and Asia were again recognized as the most unstable based on these statistics.

**Figure 3:**
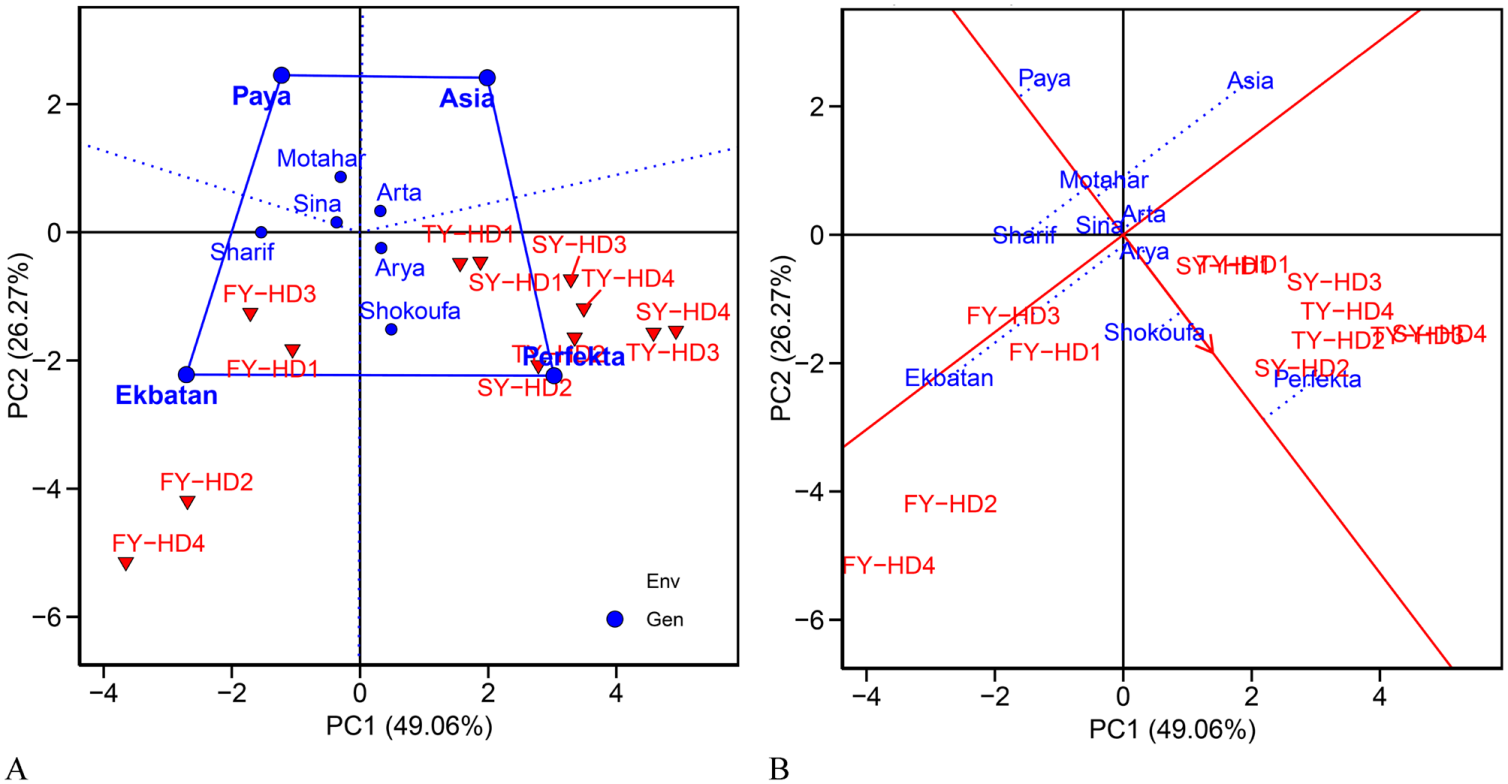
(**A**) Polygon generated through the GGE biplot method to identify optimal cultivars for each environment, and (**B**) Ranking of cultivars based on average white sugar yield and stability. *FY* first year, *SY* second year, *TY* third year, *HD*_*1*_ harvest dates on August 13, *HD*_*2*_ harvest dates on September 7, *HD*_*3*_ harvest dates on October 3, *HD*_*4*_ Harvest dates on October November 12. (**B**) The red axis featuring an arrow and intersecting the coordinate origin symbolizes stability (AEC), while the red axis marked solely by a line denotes the average yield of the genotypes.

Figure 3A and B depicts the convex hull generated by the GGE biplot analysis of sugar beet cultivars across 12 environments, utilizing the IPC_1_ and IPC_2_ to identify cultivars and environments. The diagram, accounting for 75.33% of the variance in the GEI, illustrates those cultivars closer to a specific environment exhibit specific adaptability, while those nearer to the coordinate origin display general adaptability. The study identified Sina, Arya, and Arta as the most stable due to their proximity to the coordinate origin, while Perfekta, Ekbatan, Paya, and Asia were characterized as the most unstable.

The polygon in the biplot (Fig. 3A) represents the cultivars excelling in specific environments. In this biplot, a polygon is formed by connecting the cultivars that are the furthest from the coordinate origin. The cultivars Asia, Perfecta, Ekbatan, and Paya are positioned at the maximum distances, creating the vertices of the polygon. From the coordinate origin, perpendicular lines are drawn to the sides of this polygon, delineating the mega-environments^31^. The sections where environments are placed, with cultivars positioned at their vertices, indicate that these cultivars have superior performance in those environments; in other words, they are the best cultivars for cultivation in these specific conditions. Based on this analysis, Perfecta and Shokofa were identified as the best cultivars for all four harvest dates in the second and third years, while Ekbatan was the best cultivar for all four harvest dates in the first year. Cultivars located in sections without any environments are deemed unsuitable for cultivation in any of the tested environments, categorizing them as weak cultivars. The polygonal biplot further grouped the experimental environments into two mega-environments based on WSY. The four harvest dates of the second and third years were clustered into one mega-environment, while the four harvest dates of the first year formed another. This grouping indicates that the environmental conditions of the second and third years are similar to each other, and distinct from those of the first year.

The average environment coordination line (AEC), a diagonal line passing through the biplot's center and the ideal point, indicates that genotypes closer to the circle’s center yield higher. Conversely, those further from the perpendicular line to the environmental function’s average line are less stable, exerting a more significant impact on interaction. The study highlighted Shokoufa as a cultivar with a higher average WSY and recognized its stability due to its proximity to the ACE line. In contrast, Paya exhibited the most significant distance from the ACE line, indicating weak stability compared to other cultivars (Fig. 3B).

## Discussion

The findings from this variance analysis provide profound insights into the multifaceted influences on the quantitative and qualitative traits of sugar beet, encompassing WSY, RY, SC, Na^+^, K^+^, and N. The significant effects observed across various factors highlight the intricate interplay between cultivar, environmental conditions, and agronomic practices.

The most striking result is the pronounced effect of the year on all measured traits at the 1% probability level. This underscores the critical role that environmental conditions, including climatic variables such as temperature, precipitation, and sunlight, play in determining both the yield and quality of sugar beet crops. The significant year-to-year variability in these conditions suggests that any agricultural practice or cultivar designed to optimize sugar beet production must be adaptable to fluctuating environmental conditions. This finding aligns with previous studies that have highlighted the importance of weather patterns and climate change on crop performance.

The cultivar's main effect was significant for SC at the 5% and K^+^at the 1% probability level. This indicates that genetic factors are crucial for specific traits related to sugar quality. The significant interaction between year and cultivar at the 1% probability level for all traits further emphasizes that while cultivar has a foundational impact, its expression can be profoundly modified by environmental conditions. This interaction suggests that breeding programs should focus on developing cultivars that are not only high-performing under optimal conditions but also resilient to environmental variations.

The effect of the harvest date on WSY and RY at the 5% probability level reveals that the timing of harvest is a critical agronomic practice that can influence productivity. The significant two-way interaction between harvest date and environmental conditions, as well as the three-way interaction involving cultivar, environmental conditions, and harvest date, at the 1% probability level, highlights the complexity of optimizing harvest time. These interactions suggest that the ideal harvest date may vary depending on both the specific cultivar and the prevailing environmental conditions of the year. Therefore, dynamic harvest strategies that can be adjusted based on real-time environmental monitoring may be necessary to maximize yield and quality. For breeders, these findings suggest a dual focus on genetic improvement and environmental adaptation. Future breeding programs might benefit from incorporating traits that confer stability under diverse environmental conditions. Moreover, the development of predictive models that integrate genotype performance with environmental data could facilitate better decision-making in crop management. Based on the obtained results from the experiment conducted by Sadeghzadeh Hemayati et al.^58^ the environment and its interaction with the genetic structure of different genotypes played a significant role in the phenotypic expression of WSY in sugar beet genotypes. This resulted in different responses in terms of WSY based on the conditions of different environments. Similarly, the study conducted by Saremirad and Taleghani^38^ indicated that GEI outweighs the quantitative and qualitative characteristics of sugar yield in sugar beet hybrids. Therefore, this interaction should be considered when breeding new hybrids, as it allows for decisions regarding breeding for general or specific adaptation, depending on the yield stability in different environmental conditions. In order to better understand and reveal the GEI, multivariate statistical methods can be more useful. These methods can provide insights into the complex relationships between genotypes and environments, allowing for a more comprehensive understanding of the factors influencing crop yield and the development of cultivars with stability and adaptability to target environments^35^.

The application of the MTSI offers a robust framework for evaluating the overall performance of sugar beet cultivars across multiple traits. By integrating various independent traits such as RY, SC, Na^+^, K^+^, and N, the MTSI index facilitates a holistic assessment of cultivars. This comprehensive evaluation is essential for breeding programs focused on improving multiple quantitative and qualitative traits simultaneously. In fact, its comprehensive approach facilitates the identification of top-performing cultivars, as demonstrated by the selection of Arta, Arya, and Sina in this study.

The effectiveness of the MTSI index is further validated by its successful application in previous studies across different crops and conditions. Sharifi et al.^59^ use of the MTSI index in rice genotypes to identify superior genotypes based on yield and stability highlights its versatility. Similarly, Rajabi et al.^60^ application of the MTSI index in identifying stable sugar beet genotypes under rhizomania disease conditions demonstrates its relevance in stress environments. Taleghani et al.^39^ study, which used the MTSI index to identify genotypes with desirable traits such as RY, WSY, SC, and ECS, further corroborates its efficacy. The findings of these studies align with the results obtained in this research, demonstrating the effectiveness of the MTSI index in identifying superior genotypes.

The findings from the AMMI model analysis provide a comprehensive understanding of the interactions between cultivars and environments concerning WSY. The significant additive effects of cultivars and environments indicate that both genetic makeup and environmental conditions independently contribute to variations in WSY. However, the significant multiplicative effects (GEI) reveal that the interaction between cultivar and environment is also crucial. AMMI model indicated that the GEI was significant and 2.77 times larger than the cultivar effect. This finding is consistent with several studies that have recorded a significant GEI effect in sugar beet field trials^16,35–37,39,61^. Environmental variables such as temperature, solar radiation, precipitation, and soil properties play a crucial role in determining where and how plants grow^38,62^. Weather conditions during the trial varied significantly, with extreme drought and exceptionally high temperatures observed in the first year, while the second and third years experienced adequate rainfall and good distribution. The study noted that the absence of rainfall and lower temperatures in April of the first year resulted in fewer plants per unit area. An important reason for the GEI is likely due to source limitation in most growth phases of sugar beet^12^. This finding is important for breeders and farmers as it suggests that selecting genotypes solely based on their average performance might not be sufficient; instead, their performance stability across various environments should also be considered.

The use of biplots to visualize the interaction effects provides practical insights into the stability and adaptability of different sugar beet cultivars. Cultivars like Shokoufa and Arya, which exhibit high WSY and low IPC_1_ values, are identified as desirable due to their stability across different environments. This makes them suitable candidates for regions with variable growing conditions. On the other hand, cultivars such as Ekbatan and Perfekta, with high IPC_1_ values, demonstrate greater sensitivity to environmental variations, indicating lower stability. This information is crucial for breeding programs aimed at developing robust cultivars that can withstand environmental variability. Ebmeyer et al.^34^ studied six sugar beet genotypes in eight locations over 2 years. They found significant effects of GEI on RY and SC. In addition, they indicated that high yield potential did not ensure sustainable high yields.

As can be seen in Fig. 2A across all 3 years of the experiment, the highest WSY was obtained from late harvest dates. The highest WSY was recorded by HD_4_ in the first year and HD_3_ in the second year. In the third year, the highest WSY was obtained from HD_4_. The study recommended harvesting on either HD_4_ or HD_3_ to achieve the highest WSY, as these times were found to be ideal for maximizing WSY. The higher WSY and its components in HD_4_ and HD_3_ were attributed to their longer growth period, better utilization of environmental factors such as light, temperature, and humidity, and synchronization of growth stages with favorable environmental conditions. Previous research has suggested that shortening the growth period reduces WSC and subsequently decreases RY^63^. Other investigations have also indicated the potential benefits of delaying root harvest for sugar beet cultivation^14,16,64,65^.

The identification of cultivars such as Aria and Motahar, which are close to the origin in the biplot of the first and second IPCs, indicates their general adaptability. These cultivars show less fluctuation in yield across different years and harvest dates, making them reliable choices for consistent production. The ability to identify such generally adaptable cultivars is vital for ensuring stable agricultural output in the face of changing climatic conditions.

The AMMI model's stability statistics—ASTAB, AVAMGE, DA, DZ, EV, FA, and SIPC—are pivotal in determining the stability of cultivars. Arya and Arta, which had the lowest values for these statistics, emerged as the most stable cultivars. Their low values indicate minimal interaction with environmental variables, making them reliable choices for diverse growing conditions. Conversely, Ekbatan and Asia, with the highest values, were identified as the most unstable cultivars, implying significant GEIs and less predictable performance. Further stability metrics, including ASI, ASV, MASI, MASV, Za, and WAAS, corroborated the findings of AMMI model. Motahar and Arya's low values in these metrics reinforced their status as the most stable cultivars. The consistency of Arya across multiple stability metrics underscores its robustness and adaptability. Once again, Ekbatan and Asia were identified as the least stable, highlighting their susceptibility to environmental variability.

The identification of stable cultivars like Arya and Arta is particularly valuable for breeding programs. These cultivars can serve as foundational genotypes for developing new cultivars that combine high yield with stability. The dual focus on yield performance and stability ensures that the resulting cultivars are not only productive but also resilient to environmental fluctuations. The findings suggest that incorporating stability metrics into the selection criteria can significantly enhance the effectiveness of breeding programs.

## Conclusion

In the spring cultivation of sugar beet, delaying the harvest can increase WSY, but it may also hinder timely field preparation for the next crop due to autumn rains. Therefore, identifying the optimal harvest dates is crucial to maximize economic yield and ensure adequate time for land preparation. This research found that the highest WSY was achieved with harvest dates HD_4_ and HD_3_. However, harvesting on HD_3_ is recommended to balance yield and field preparation time. Selecting the genotype with the highest WSY would be advantageous across all harvest dates, allowing for efficient selection during the first harvest and reducing time and costs. Notably, Perfekta demonstrated the highest WSY over 3 years, followed by Shokoufa. When assessing cultivar stability based on WSY, Arta and Arya showed high general stability, while Perfekta and Ekbatan exhibited high specific stability. Furthermore, the MTSI identified Arta, Arya, and Sina as the most stable cultivars. These cultivars emerged as the top-ranking cultivars, suggesting them as a potential candidate for further breeding programs.

## Data Availability

The data that support the findings of this study are available from the corresponding author upon reasonable request.
